# Engineered Orange Ectopically Expressing the Arabidopsis β-Caryophyllene Synthase Is Not Attractive to *Diaphorina citri*, the Vector of the Bacterial Pathogen Associated to Huanglongbing

**DOI:** 10.3389/fpls.2021.641457

**Published:** 2021-03-02

**Authors:** Berta Alquézar, Haroldo Xavier Linhares Volpe, Rodrigo Facchini Magnani, Marcelo Pedreira de Miranda, Mateus Almeida Santos, Viviani Vieira Marques, Márcia Rodrigues de Almeida, Nelson Arno Wulff, Hieng-Ming Ting, Michel de Vries, Robert Schuurink, Harro Bouwmeester, Leandro Peña

**Affiliations:** ^1^Laboratório de Biotecnologia Vegetal, Pesquisa & Desenvolvimento, Fundo de Defesa da Citricultura (Fundecitrus), Araraquara, Brazil; ^2^Instituto de Biología Molecular y Celular de Plantas (IBMCP), Consejo Superior de Investigaciones Científicas (CSIC), Universidad Politécnica de Valencia (UPV), Valencia, Spain; ^3^Chemistry Department, Universidade Federal de São Carlos (UFSCar), São Carlos, Brazil; ^4^Institute of Chemistry, São Paulo State University (UNESP), Araraquara, Brazil; ^5^Institute of Plant Biology, National Taiwan University, Taipei, Taiwan; ^6^Swammerdam Institute for Life Sciences, Green Life Sciences Cluster, University of Amsterdam, Amsterdam, Netherlands

**Keywords:** Asian citrus psyllid, biotechnology, HLB, sesquiterpenes, chemical ecology, *Citrus sinensis*, transgenic, volatiles

## Abstract

Huanglongbing (HLB) is a destructive disease, associated with psyllid-transmitted phloem-restricted pathogenic bacteria, which is seriously endangering citriculture worldwide. It affects all citrus species and cultivars regardless of the rootstock used, and despite intensive research in the last decades, there is no effective cure to control either the bacterial species (*Candidatus* Liberibacter spp.) or their insect vectors (*Diaphorina citri* and *Trioza erytreae*). Currently, the best attempts to manage HLB are based on three approaches: (i) reducing the psyllid population by intensive insecticide treatments; (ii) reducing inoculum sources by removing infected trees, and (iii) using nursery-certified healthy plants for replanting. The economic losses caused by HLB (decreased fruit quality, reduced yield, and tree destruction) and the huge environmental costs of disease management seriously threaten the sustainability of the citrus industry in affected regions. Here, we have generated genetically modified sweet orange lines to constitutively emit (*E*)-β-caryophyllene, a sesquiterpene repellent to *D. citri*, the main HLB psyllid vector. We demonstrate that this alteration in volatile emission affects behavioral responses of the psyllid in olfactometric and no-choice assays, making them repellent/less attractant to the HLB vector, opening a new alternative for possible HLB control in the field.

## Introduction

Huanglongbing (HLB) is currently considered the most serious disease of citrus as it is devastating citriculture almost worldwide (reviewed in Bove, 2006; Gottwald, 2010; da Graça et al., 2016; Zheng et al., 2018). The disease is caused by phloem-limited bacteria (*Candidatus* Liberibacter spp.: *Candidatus* L. asiaticus, *Candidatus* L. americanus, and *Candidatus* L. africanus; *C*Ls) vectored by the sap-sucking psyllids *Diaphorina citri* Kuwayama (Hemiptera: Liviidae) and *Trioza erytreae* (Del Guercio) (Hemiptera: Triozidae). HLB-infected trees decline in productivity and fruit quality and, in aggressive situations, even die within a few years, inducing severe economic losses to the growers (Bassanezi et al., 2009). In addition, current HLB management is based on removing symptomatic trees and reducing psyllid vector population by frequent insecticide treatments, which boost production costs. For example, since HLB detection in Florida (United States) in 2005, millions of trees have been killed, production volume has decreased by more than 74%, and annual economic losses are estimated at hundreds of million $ (Hodges et al., 2014; Singerman and Rogers, 2020). Despite intensive chemical control programs, HLB infection, which before 2004 was restricted to Asia, Eastern Africa, and the Arabic Peninsula, is currently present in all major citrus-producing areas except Australia/New Zealand and the Mediterranean basin.

From the mid-2000s, when HLB was detected in Brazil and the United States—the second and third largest worldwide citrus producers, respectively—research endeavors aimed at finding a cure for the disease as well as limiting its expansion increased tremendously. In the United States alone, more than 545 million dollars have been invested in research to control HLB since 2009 (Belasque et al., 2010; National Academies of Sciences, Engineering, and Medicine, 2018). As a result, there is a lot of information regarding *D. citri* biology (the vector present in the Americas). Notwithstanding these tremendous research efforts, the causal bacteria can still not be cultured, and HLB remains incurable. Consequently, the best management strategy to control HLB spread and infection is, as for any vector-borne pathogen transmitted in a circulative manner, based on two essential actions: (1) diminish the amount of psyllids able to spread the bacterium, and (2) reduce the quantity of inoculum accessible to the vectors (National Academies of Sciences, Engineering, and Medicine, 2018). With regard to the former, both nymphs and adults from *D. citri* are able to acquire the bacterium while feeding on infected plants and transmit it to healthy plants when feeding on them. Although eco-friendly control strategies, such as essential oils, botanical insecticides, plant-derived semiochemicals, and biological control, have been investigated (Khan et al., 2014), the reality is that psyllid population management still relies mostly on the use of numerous and aggressive insecticide applications. To reduce the number of chemical applications, large area-wide management (10,000–50,000 acres), which has been shown to increase the effectiveness of the treatments and reduce management costs, is recommended (Bassanezi et al., 2013; Boina and Bloomquist, 2015). Despite this, chemical control remains costly, unsustainable, and not fully effective (Hall et al., 2013; Yan et al., 2015). Besides, in less than a decade, *D. citri* has developed resistance to a number of insecticides (Tiwari et al., 2012).

*Candidatus* Liberibacter asiaticus (*C*Las)-infected leaves become infectious for psyllids within only 15 days, but HLB symptoms appearance takes months to years (Lee et al., 2015). This circumstance greatly complicates the elimination of inoculum reservoirs, which is mainly based on the visual identification of early HLB symptoms, infection confirmation by molecular techniques, and subsequent elimination of infected trees. Even so, a three-pronged system (TPS) based on (i) psyllid population control, (ii) removal of infected trees, and (iii) use of healthy citrus plants from certified nurseries is up to date the best management strategy to limit HLB spread in the three major citrus-producing countries worldwide, China, Brazil, and United States (Belasque et al., 2011; Bové, 2012; Gottwald et al., 2012; Zheng et al., 2018).

Because an efficient breakthrough to stop HLB infection and psyllid spread is still lacking, there is a need to adopt new alternative environmentally friendly pest management approaches to sustain economic viability of the citrus industry. *D. citri* is oligophagous on rutaceous host plants, including all *Citrus* species and related genera. Up to date, no source of resistance has been identified within *Citrus*, although different levels of susceptibility and sensitivity to the vector and/or to HLB infection have been reported within the *Rutaceae* (Nava et al., 2007; Westbrook et al., 2011; Richardson and Hall, 2013; Ramadugu et al., 2016; Hall et al., 2017; Fancelli et al., 2018; Killiny et al., 2018). If resistance traits are confirmed in relatives that are sexually compatible with citrus, generation of new resistant commercial cultivars by conventional breeding could be addressed. However, complex genetics and reproductive biology of citrus, including high heterozygosity, cross and self-incompatibility, and facultative apomixis, joined to their long juvenile period, made this approach unviable for most citrus cultivars and when attainable, extremely time consuming. In addition, the original desired features from already existing and well-known commercial varieties will likely never be fully recovered. Genetic modification of citrus opens the possibility of, once genes of resistance to the disease are identified (from sexual compatible but also unrelated genera), introducing them into elite cultivar genetic backgrounds without modification of the rest of their excellent agronomic traits. Thus, genetic modification has been pointed out as the only solution to stop this devastating disease (Bové, 2012; Duran-Vila et al., 2014; Yan et al., 2015; Mccollum and Baldwin, 2016; National Academies of Sciences, Engineering, and Medicine, 2018; Cochrane and Shade, 2019; Wang, 2020). In parallel to the identification and evaluation of traits that confer resistance—through introduction into desired genotypes and field testing—alternative environmentally safe strategies need to be developed to avoid HLB spread or to limit it as much as possible.

In the last decade, it has been shown that volatile organic compounds (VOCs) play a pivotal role in the relation of plants with organisms in its environment, including con-specifics, insects, other herbivores, and microorganisms (Yoneya and Takabayashi, 2014; Stenberg et al., 2015). Insect herbivory could be reduced by increasing the crops’ endogenous resistance traits, such as deterrence, that may have been lost during crop domestication (Beck et al., 2018). For example, maize-domesticated genotypes that differ in their VOC profiles may exhibit different levels of tolerance/resistance to pests/pathogens (Kollner et al., 2008). Alteration of odorscape by intercropping non-host plants that release different volatiles or by releasing synthetic sex pheromones from devices can alter pests’ behavior and reduce herbivory damage (reviewed in Conchou et al., 2019). Main disadvantages of these approaches are reduction of the area used with commercial-relevant crop and limitations in volatile diffusion technologies. By the other hand, it has been demonstrated that altering the VOC composition by metabolic engineering can modify the behavioral response of a pest to a host (reviewed in Pickett and Khan, 2016). For example, engineered wheat overproducing (*E*)-β-farnesene repelled cereal aphids (Bruce et al., 2015). More recently, it has been described that overexpression of *OsTPS46* in rice leads to increased emission of D-limonene and (*E*)-β-farnesene, by which the aphid *Rhopalosiphum padi* is deterred (Sun et al., 2017). Furthermore, *OsTPS46* silencing turns rice vulnerable to *R. padi*, which does not usually attack it (Sun et al., 2017). Similarly, *Arabidopsis* plants overproducing β-patchoulene repelled beet armyworm feeding activity (Pu et al., 2019). Thus, it seems that modulation of VOCs directed to disrupt pathogen–host plant interactions is a viable approach for pest management that would have no residual effects on the environment. Psyllid species rely on olfactory cues to find their hosts, but evidences are difficult to obtain by laboratory bioassays, as physiological stage, light intensity, and the presence of visual cues influence their behavior (Nissinen et al., 2008; Wenninger et al., 2009; Farnier et al., 2018). However, different studies indicated that psyllids respond positively to their host odors, for example, the common pistachio psyllid *Agonoscena pistaciae* is attracted to volatiles of pistachio leaves (Ghamari et al., 2018) and *Cacopsylla pruni* to those of apricot (Andrianjaka-Camps et al., 2018). In this last work, by simultaneously analyzing volatile profiles and psyllids response to different apricot cultivars, putative attractive and repulsive compounds to *C. pruni* were identified. Repellent volatiles to the plum psyllid *C. pruni* have been also identified from plum (Gallinger et al., 2019). Some non-host volatiles, as dimethyl-disulfide (DMDS), have been proven to be repellent to the potato psyllid *Bactericera cockerelli* (Diaz-Montano and Trumble, 2013) and to the psyllid *Apolygus lucorum*, an important cotton pest (Pan et al., 2013). Because of the great impact that HLB is causing to the citrus industry, there is much more information available on the *D. citri*/Rutaceae pathosystem, which suggests that this strategy may be implemented to control *D. citri* infestation and, consequently, *C*Ls infection. *D. citri* host plant selection is mediated by olfactory perception of volatile compounds (Patt and Sétamou, 2010) as well as by visual cues (Wenninger et al., 2009; Miranda et al., 2015). Furthermore, *D. citri* can discriminate between different host blends (Fancelli et al., 2018) and even between different qualitative conditions of host plants (Mann et al., 2012). Besides, host attractiveness can be disrupted by the presence of repellent volatiles, as was demonstrated for those of guava leaves (Rouseff et al., 2008; Zaka et al., 2010; Silva et al., 2016).

Recently, we showed that (*E*)-β-caryophyllene emission could turn *Arabidopsis*, a neutral non-host plant species, to become *D. citri* repellent, and that this effect was maintained even in a background of citrus volatiles, envisaging the potential of this strategy to control HLB spread (Alquézar et al., 2017b). To further explore this approach, we decided to engineer Valencia sweet orange to constitutively emit (*E*)-β-caryophyllene. The resulting transgenic lines emitted large amounts of β-caryophyllene, without other remarkable differences in their phenotype. This feature made the usually highly attractive sweet orange flushes non-attractive to *D. citri*, as shown by olfactometric behavioral assays, opening up a new potential alternative strategy to control HLB.

## Materials and Methods

### Generation and Southern Blot Analysis of Transgenic Sweet Orange Lines Expressing *AtCS* Under the Control of a Constitutive Promoter

A previously available binary PinPlus vector containing a 2,160-bp genomic clone of *Arabidopsis thaliana* (*E*)-β-caryophyllene synthase (*TPS21*, *At5g23960*) under the control of CaMV35S promoter and Rbsc 1 terminator (Ting et al., 2015) was transferred to *Agrobacterium tumefaciens* EHA105 (Supplementary Figure 1). Transformation of stem segments from adult plants of sweet orange (*Citrus sinensis* L. Osb cv. Valencia) was performed as described previously using kanamycin at 75 mg/L as selection pressure (Rodríguez et al., 2008). Genomic DNA from regenerated explants was extracted using CTAB method (McGarvey and Kaper, 1991), and the presence of the T-DNA was checked by PCR using primers 35S-F (5′-CACAATCCCACTATCCTTCG-3′) and B137 (5′-CGTACTATGCTTCTCTTTG-3′). All PCR-positive shoots were grafted *in vitro* onto “Troyer” citrange (*Citrus X sinensis* L. Osb. X *Poncirus trifoliata* L. Raf.) seedlings. Approximately 3 months later, the plantlets were grafted in a greenhouse into 5-month-old “Rangpur” lime (*Citrus X limonia* Osb.) rootstocks potted in coconut fiber.

For Southern-blot analysis, gDNA was isolated from young leaves as previously described (Dellaporta et al., 1983) and subjected to overnight digestion with *Nhe*I or *Nhe*I and *Sac*I restriction enzymes (NEB) to determine loci number and integrity of the inserted T-DNA, respectively. Digested DNA was resolved on a 1% agarose gel for 16 h at 40 V and blotted onto nylon membranes (Hybond-N+, Amersham Pharmacia) by overnight capillary transfer. Blots were hybridized with a digoxigenin-labeled (DIG-11-dUTP; Roche Diagnostics) fragment of the *nptII* gene and detected by chemiluminescence according to manufacturer’s instructions (Roche Diagnostics).

### Quantitative RT-PCR Analysis

Total RNA was extracted from young flushes and treated with DNase using RNeasy mini kit (Qiagen). cDNA synthesis was carried out using oligo dT(18) and RTII (Invitrogen, Thermo Fisher Scientific, United States). qRT-PCR was run on a StepOne Plus Real Time PCR System (Applied Biosystems, United States) using SYBR Green PCR Master Mix (Applied Biosystems, United States) and 50 ng of total cDNA. The primers used to investigate *AtCS* expression were AtCSF (5′-GCATGGGGAGTGAAGTCAAC-3′) and B137 (5′-CGTACTATGCTTCTCTTTG-3′). Citrus *GAPC2* and *ACTIN* genes were used as housekeeping reference genes (Mafra et al., 2012). Expression level of *AtCS* was determined as described by Livak and Schmittgen (2001) and normalized to the average *AtCS* expression level of all samples. Values are presented as the mean ± SD of at least three independent analyses. Statistical analyses were performed using ANOVA (LSD test, *p* < 0.01).

### Phenotypic Analysis

Selected transgenic lines were propagated by grafting onto Rangpur lime rootstocks to generate at least 15 independent clones for each genotype of interest. Plants were grown under greenhouse conditions for 18 months without detecting any detrimental effect on development and growth. At this moment, phenotypic evaluations were performed. Twelve leaves were taken from eight independent plants from each selected line, and their foliar surface was measured employing area integrator equipment (LiCor 3100 C; LI-COR Inc., Lincoln, NE, United States). The length and the diameter of the first 50 internodes were measured using a ruler and digital Vernier caliper, respectively, at 10 different points along the stalk of at least eight independent plants for each evaluated line measured.

Fruit quality parameters were measured on three biological replicates, each of them of at least 10 independent fruits from eight independent plants for each line. Diameter was measured on the equatorial plane using a MITUTOYO digital caliper (Illinois, United States). Weight was estimated using a regular bascule. L, a, and b parameters were determined using a Minolta CR-200 Chroma Meter (Osaka, Japan), and color index (CI) was expressed as 1,000 a/L⋅b. Juice quality analyses were performed by JBT Research Technology Center for Fruit and Vegetable Processing (Araraquara, Brazil) with one box (40.8 kg) of fruit per line. Juice acidity was determined according to AOAC methods by titration with 0.1 N NaOH, using phenolphthalein as a visual end-point indicator, and expressed as the percentage of anhydrous citric acid by weight. The soluble solid content (Brix) was estimated by refractometry, using an Atago^®^ refractometer. The maturity index is expressed as the ratio of Brix/acidity.

### Volatile Organic Compound Content Extraction and Analysis

For each genotype of interest, plant material was obtained from six independent clones, making three different pools of young flushes from two different plants for each studied line. At least three extractions for each biological replicate were performed. Flushes were collected, frozen, and homogenized in liquid nitrogen and stored at −80°C until extraction. One milliliter of hexane supplemented with 1.0 μg of *n*-eicosane was added to 50 mg of frozen ground material, vortexed for 5 min, and centrifuged at 9,800 × *g* for 5 min at 4°C. Upper phase was recovered on a new tube, and aliquots of 0.5 μl were introduced in a split ratio adjusted to 20:1 into the injection port, maintained at 250°C. A gas chromatograph coupled to a mass spectrometer (GCMS-QP2010 Plus, Shimadzu, Corporation, Kyoto, Japan) and equipped with a dimethyl polysiloxane capillary column (Rxi^®^-5 ms 10 m × 0.10 mm i.d. × 0.10 μm film; Restek Corporation, Bellefonte, PA, United States) was used for volatile content analysis. The column temperature was kept at 40°C for 1 min, ramped at 30°C min^–1^ to 150°C, then increased to 10°C min^–1^ to 180°C, and finally ramped at 70°C min^–1^ to 250°C and held at this temperature for 1 min. The detector interface and the ion source temperatures were 250 and 200°C, respectively, and the carrier gas was He (0.45 ml min^–1^). Mass spectra were recorded at 70 eV with a mass scan range from *m/z* 40 to 500. Total peak areas were quantified in the total ion chromatogram (TIC) and normalized to the recovery rate of the internal standard (*n*-eicosane). Compounds were identified by matching of the acquired mass spectra with those stored in reference libraries (NIST and Wiley) and databases from retention index, related to *n*-alkanes (C_8_–C_20_, Sigma–Aldrich). The relative amounts of individual terpenes are referred to the mean area quantified in the four EV control lines analyzed, to which was assigned an arbitrary value of one.

### Volatile Organic Compounds Emission Analysis and Quantification

To collect emitted volatiles, young flushes from independent plants were enclosed in oven bags cinched at 500-ml volumes with plastic ties. A constant charcoal-filtered humidified air flow (0.3 l min^–1^) was passed through the container, and the outgoing stream was directed to 0.635-cm-diameter polytetrafluoroethylene (PTFE) tubes (Sigma–Aldrich, Bellefonte, PA, United States) filled with 200 mg of Tenax^®^-TA, 35–60 mesh (Sigma–Aldrich, Bellefonte, PA, United States). Plants were maintained at constant temperature (24–25°C), relative humidity (60–73%), and L14:D10 photoperiod (3,000 lux), while volatiles were continuously collected for 24 h. Tenax cartridges were eluted with 3 × 500 μl of pentane:diethyl ether (80:20 v/v) supplemented with 50 pg/μl of tetralin (Sigma–Aldrich) to be used as internal standard.

The sample aliquots (1 μl) were injected at 50°C into a gas chromatograph (Agilent 7890A) coupled to an accurate-mass quadrupole time-of-flight mass spectrometer (Agilent 7200). The injector port was heated to 275°C at a rate of 240°C per min. The oven temperature was maintained at 40°C for 3 min and increased by 5°C per min until it reached 140°C. Then it was heated at a rate of 10°C per min to 250°C, and maintained at this temperature for 5 min. The equipment was operated in electron-impact mode, and mass spectra were recorded at 70 eV with a mass scan range from *m/z* 40 to 500. Compounds were separated on a capillary HP-5 ms column (30 m × 250 μm, 0.25 μm film thickness; Agilent) with helium as the carrier gas at a flow rate of 1 ml/min^–1^. Available commercial terpene standards were used for compound identification and quantification for most compounds. Camphene, (*E*)-β-ocimene, and α-copaene were identified by spectral match on reference libraries and Kovats index, and quantified employing sabinene, myrcene, and (*E*)-β-caryophyllene quantification curves, respectively.

### Insect Rearing

*C*Las-free and *C*Las-infected psyllids continuously reared cultures are maintained in climatic-controlled rooms at 25 ± 2°C, 65 ± 10% RH, and L14:D10 photoperiod (3,000 lux) at Fundecitrus (Araraquara, Brazil). Healthy *D. citri* individuals are maintained on *Murraya paniculata* (L.) Jack seedlings. Infected psyllids were obtained by keeping them during their whole entire life cycle (eggs, nymphs, and adults) on *C*Las-infected “Valencia” sweet orange nursery trees grafted on Rangpur lime, as described by Carmo-Sousa et al. (2020). These “Valencia” plants were *C*Las-infected by grafting budwood from a previously *C*Las infected plant, and positive bacterial infection was confirmed before psyllids rearing by qPCR, as described before (Li et al., 2006).

### *Diaphorina citri* Olfactometric Assays

*Diaphorina citri* responses to volatiles emitted by CS and EV lines were evaluated in a four-arm olfactometer essentially as described by Volpe et al. (2020). In brief, air-flows of 0.1 ml min^–1^ were passed through bagged flushes or empty bags and directed to olfactometer fields. At each replicate, a single 7–15-day-old mated *D. citri* female (*C*Las-infected or not), drawn from continuously reared cultures, was released in the middle of the arena, and its behavior to the different odor fields (two from plants, two of clean air) was evaluated. Mated and single-gender females were used to avoid any conspecific volatile that could alter psyllid behavior (e.g., sex pheromones) and because they are more responsive to citrus volatiles than males (Volpe et al., 2020). The time spent in each odor source was evaluated for 10 min. Psyllids that did not perform a choice after 5 min were recorded as “not responsive.” For each genotype, response of the psyllid was evaluated at 10 different days, with at least nine responsive psyllids per day. Time spent in each odor source (plant or clean air) for each insect was expressed as percentage. All experiments were conducted at 25 ± 1°C, 65 ± 5% RH, and constant light (3,000 lux).

After olfactometry assays with *C*Las-infected psyllids, each tested female was processed in a Tissue Lyzer (Quiagen, Germany) using stainless steel beads and their DNA extracted (Murray and Thompson, 1980). DNA from each single psyllid was analyzed for the presence of *C*Las (Li et al., 2006) and *D. citri* DNA (Manjunath et al., 2008). All tested psyllids were *C*Las-positive (Ct values ≤ 35.0 for HLBaspr).

### Choice Behavioral Test

A no-choice test was designed to evaluate the effect promoted by volatiles emitted by orange CS and EV plants. Transparent acrylic boxes (55 × 40 × 35 cm in length, width, and height, respectively) with a rectangular acrylic lid (63 × 48 4 cm in length, width, and height, respectively) were constructed (Supplementary Figure 3). Two holes (7.5 cm in diameter) were made on two opposite sides (length size) of each box (one in each side). In one side of the box, a central processing unit (CPU) fan cooler connected to a digital potentiometer was coupled on a hole covered with veil tissue. On the opposite side, a black PTFE cylinder was fixed on another hole (same size used to connect the fan) and used as a platform to release the psyllids. The bottom of the cylinder was covered with a veil tissue. A small hole was made in the cylinder to release psyllids into the device and covered with a cylindric lid. A third hole (same diameter used for the other two) was made on the bottom of the acrylic box in order to allow the introduction of a sweet orange plant grafted onto Rangpur lime containing three flushes (10–18 cm each). In this way, the fan allowed the induced air to pass through the internal part of the box carrying on the plant volatiles to the psyllid direction, and the plume leaved the box by the bottom of the black cylinder (insect release platform). The fan speed was promoted by setting up the potentiometer to 10% of the total RPM of the fan. Before testing the repellent effect induced by citrus plants’ VOCs emission, the plants were sprayed twice until runoff with 3% kaolin concentration (Surround WP^®^; 99.4% active ingredient; Tessenderlo Kerley Inc., Phoenix, AZ, United States) using a backpack sprayer (Brudden^®^ SS 5 L; Brudden Equipamentos Ltda., Pompéia, Brazil). Ten 7–15-day-old mated females were released inside the release platform, and 10 replications were evaluated (each box was considered a replicate). The number of settled psyllids on citrus plants was assessed at 1, 4, 8, and 24 h after the insect’s release. The experiment was performed under the same room conditions (temperature, photoperiod, and relative humidity) described for olfactometer tests.

### Statistical Analyses

Data from volatile content and emission and phenotypic analysis met the assumption of the normal model, so they were submitted to a variance analysis (ANOVA) and the means were compared by LSD test. Data from olfactometric assays related to time spent in the different odor fields were processed in the same way. Measurements from no-choice behavioral experiments did not conform to simple variance assumptions. Then they were subjected to logistic regression and Chi squared (χ^2^) analysis tests. Analyses were performed using the statistical software STATGRAPHICS Centurion XVII (version 17.2.00).

## Results

### Obtainment of Transgenic Sweet Orange Plants Overexpressing *AtCS*

**FIGURE 1:**
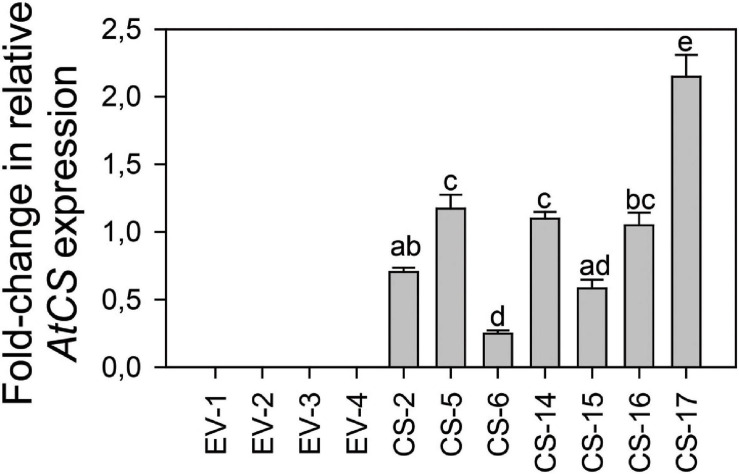
*AtCS* relative expression level in flushes of plants from different engineered sweet orange lines overexpressing *AtCS* (CS) vs. empty-vector (EV) transgenic lines after 18 months of cultivation in the greenhouse. Bars represent mean values ± SD of at least three biological replicates and different superscript letters indicate significant differences (*p* < 0.01). The average relative expression level of all CS samples was normalized to 1.

To obtain constitutive synthesis of (*E*)-β-caryophyllene, a T-DNA harboring the genomic sequence of *A. thaliana* (*E*)-β*-CARYOPHYLLENE SYNTHASE TPS21* (*AtCS*) under the control of the 35S promoter plus the *nptII* selectable marker gene cassette was constructed. This T-DNA was transferred into the genome of Valencia sweet orange (*Citrus X sinensis* L. Osb.) via *Agrobacterium*-mediated transformation of the cut ends of internodal stem tissues. A total of 10 putatively transgenic (*E*)-β*-CARYOPHYLLENE SYNTHASE* overexpressing lines (CS lines) were produced by regeneration on kanamycin-containing medium. Seven independent transgenic lines harboring a full-length single copy of the transgene in Southern blots were selected for further analysis (Supplementary Figure 1). *AtCS* expression was evaluated in leaves from young shoots, the most attractive leaf stage to *D. citri* and thus the most interesting target tissue to induce (*E*)-β-caryophyllene emission. Transcripts of the transgene were detected in all the CS orange lines analyzed, while they were absent in flushes from EV control lines (Figure 1). Lines CS-5, -14, and -17 showed comparable intermediate expression levels, while CS-2, -6, and -15 presented lower transcript amounts. Line CS-16 exhibited the highest level of *AtCS* expression, over eightfold higher than that of CS-6, and approximately doubled that of intermediate lines.

### Overexpression of *AtCS* Did Not Induce Detrimental Effects in CS Transgenic Sweet Orange Plants

**FIGURE 2:**
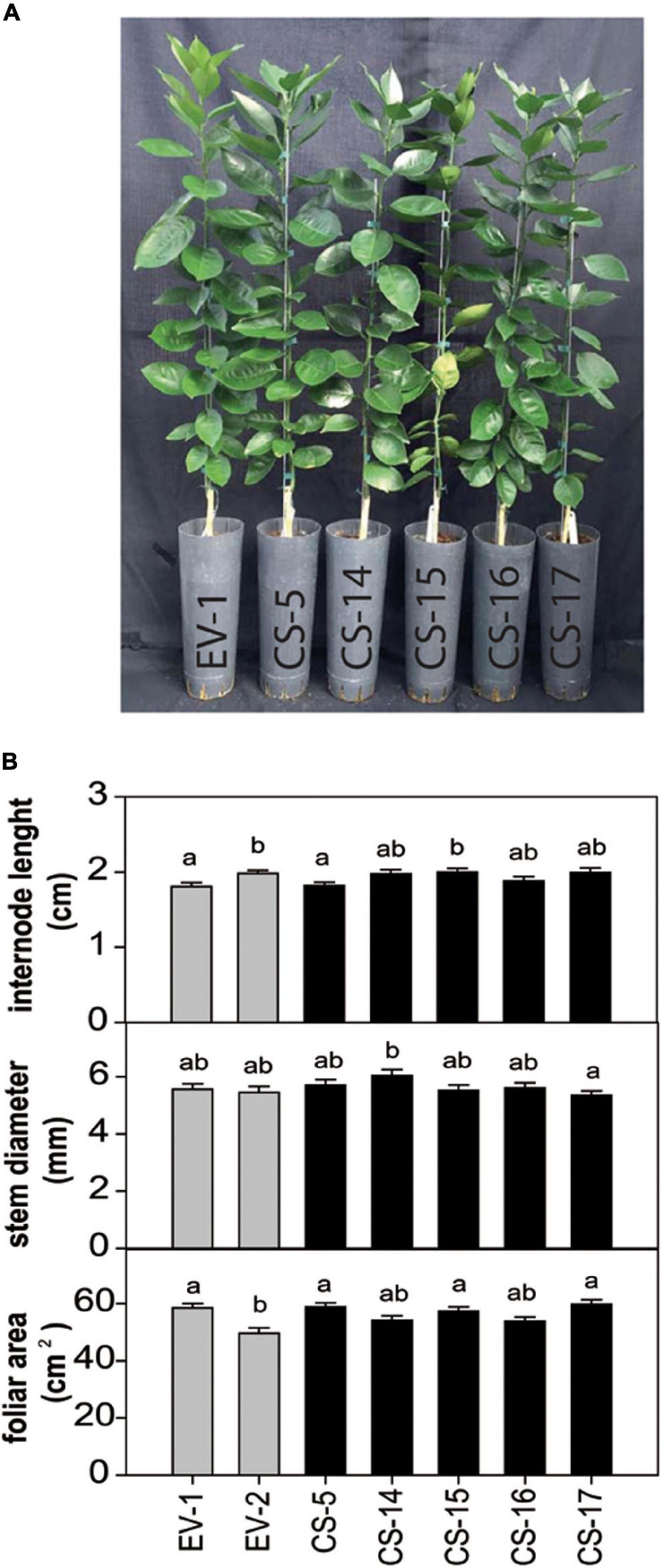
**(A)** Representative picture and **(B)** evaluation of different phenotypic characteristics in flushes of plants from selected CS and EV engineered lines after 18 months of cultivation in the greenhouse. Bars represent mean values ± SD of at least three biological replicates. Different superscript letters indicate significant differences (*p* < 0.01).

**FIGURE 3:**
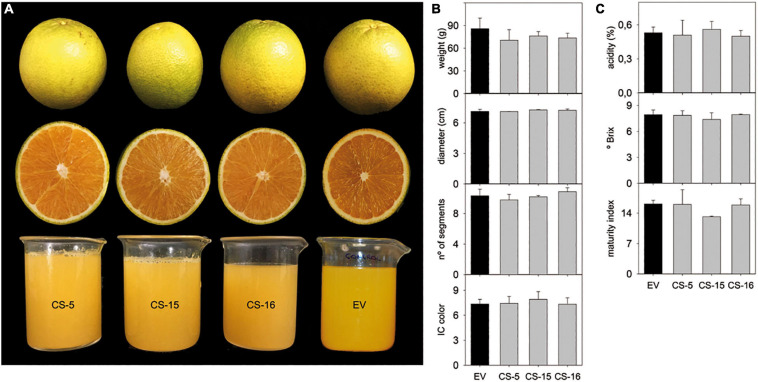
**(A)** Aspect of peel, pulp, and juice from mature fruit of EV and CS lines. Evaluation of fruit **(B)** and juice **(C)** quality parameters. Peel index color (IC) was calculated as 1,000 a/L⋅b. Bars represent mean values ± SD of at least three biological replicates. LSD-test analysis was carried out, and no statistical differences were detected.

Selected CS lines were grafted on “Rangpur” lime (*Citrus X limonia* Osb.) and propagated under greenhouse conditions without observing any detrimental effect on plant growth. The plants also showed no alteration of color or morphology, being indistinguishable at a first glance from the empty-vector (EV) control plants (Figure 2A). After 18 months, basic phenotypic characteristics, such as internode length, stem diameter, and leaf area, were evaluated. No significant differences between engineered CS- and EV-transformed lines were found (Figure 2B). To investigate whether *AtCS* heterologous overexpression induces any detrimental effect in citrus, some key fruit quality parameters were evaluated in fruits from three selected greenhouse-grown engineered lines. Visually, CS and EV fruits and juices were indistinguishable (Figure 3A). Accordingly, obtained results indicated that size, weight, number of segments, and peel color of CS fruits were statistically equal to those of EV (Figure 3B). Similarly, quality characteristics were comparable in CS and EV juices (Figure 3C). However, these results are just preliminary. Fruit setting should be evaluated for several productive seasons in the orchard to be able to get reliable results on fruit quality. Nevertheless, our first fruit evaluation was enough to claim that *AtCS* overexpression does not induce any aberrant phenotype in the GM fruits.

### Analysis of Volatile Terpene Compounds Accumulated in the Leaves of Engineered CS Sweet Orange Lines

**FIGURE 4:**
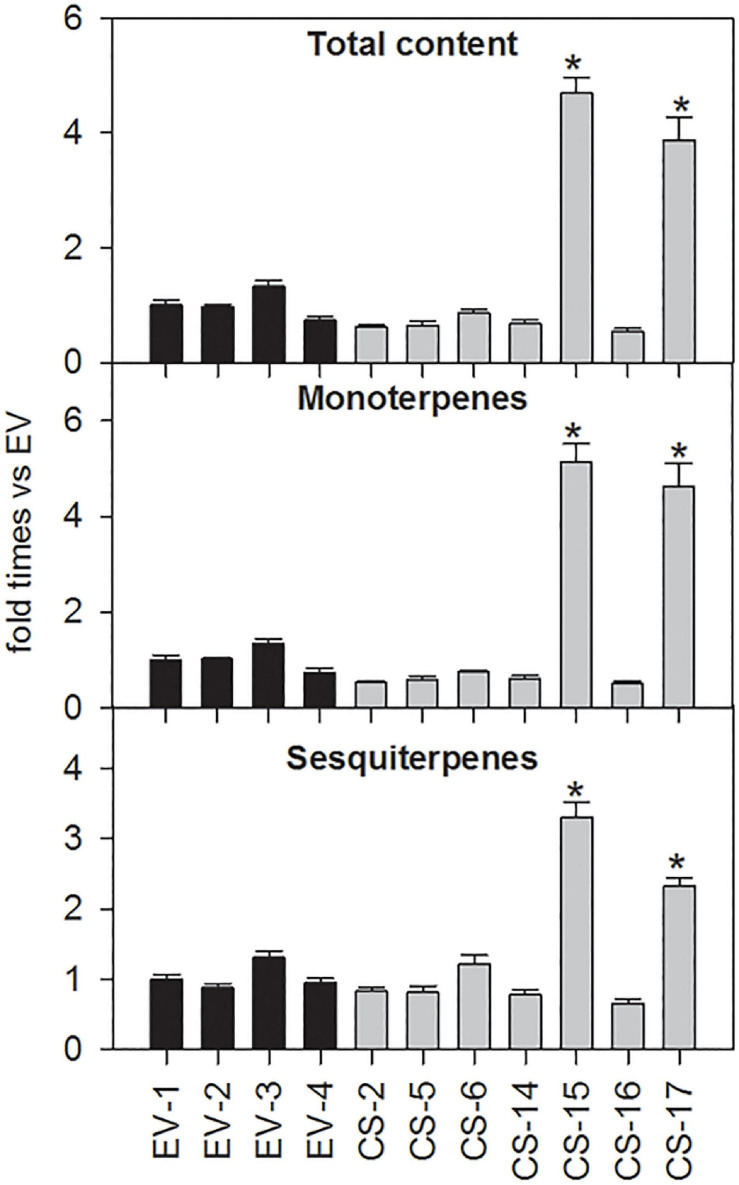
Relative accumulation of monoterpene and sesquiterpene volatile compounds in young flushes of engineered β-caryophyllene synthase (CS) lines and empty-vector (EV) controls. The data for each genotype correspond to the average ± SD of three biological replicates analyzed in three technical replicates each. For each compound, values are presented as fold change relative to the mean area value of each specific compound in all EVs analyzed. Asterisks indicate significant differences (*p < 0.01).

Terpene volatiles accumulated in young flushes of CS and EV control lines were extracted and analyzed by GC-MS. The total amount of volatile terpene compounds accumulated in the flushes of the CS lines analyzed was, in general, similar to that detected in EV controls (Figure 4). Only in CS-15 and -17, mono- and sesquiterpene content was significantly altered in relation to EVs. In these lines, monoterpene accumulation was between 4.6- and 5.1-fold higher than in EV lines, while the sesquiterpene amount was more than twofold higher than in the controls.

**FIGURE 5:**
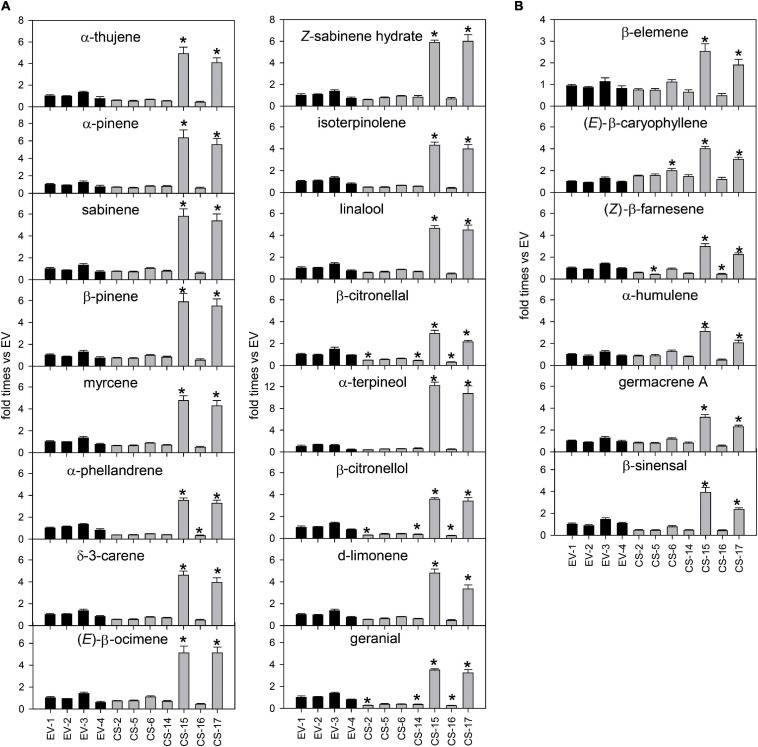
Mean ± SD difference of 14 individual monoterpenes **(A)** and six individual sesquiterpenes **(B)** accumulated in young flushes of engineered β-caryophyllene synthase (CS) lines and empty-vector (EV) controls. The data for each genotype correspond to three biological replicates analyzed in three technical replicates each. For each compound, values are presented as fold change relative to the mean area value of each specific compound in all EVs analyzed. Asterisks indicate significant differences (*p < 0.01).

The changes in the monoterpene profile were analyzed in more detail (Figure 5A). Lines CS-15 and -17 had a higher content of all the monoterpenes identified, especially α-terpineol (more than 10 times higher than EVs). The other monoterpenes, except β-citronellal, increased at least 3.5-fold relative to the controls. In CS-16, a slight decrease in the content of all the monoterpenes was detected, but to a significant extent only for α-phellandrene, β-citronellal, β-citronellol, and geranial. Lines CS-2 and -14 displayed a reduced accumulation of many monoterpene alcohols and aldehydes, of at least 50% for citronellal, citronellol, and geranial. Regarding the sesquiterpene profiles, lines CS-2 and -14 showed no significant differences when compared with EV lines (Figure 5B), while in lines CS-5 and -16, *Z*-β-farnesene content was reduced by about 60%. The largest changes were again identified in lines CS-15 and -17, which had between 1.9 (β-elemene) and four times [(*E*)-β-caryophyllene and β-sinensal] higher sesquiterpene concentrations than the EVs.

### *Diaphorina citri* Is Less Attracted to CS Than to Control Lines in Four-Arm Olfactometer Assays

**FIGURE 6:**
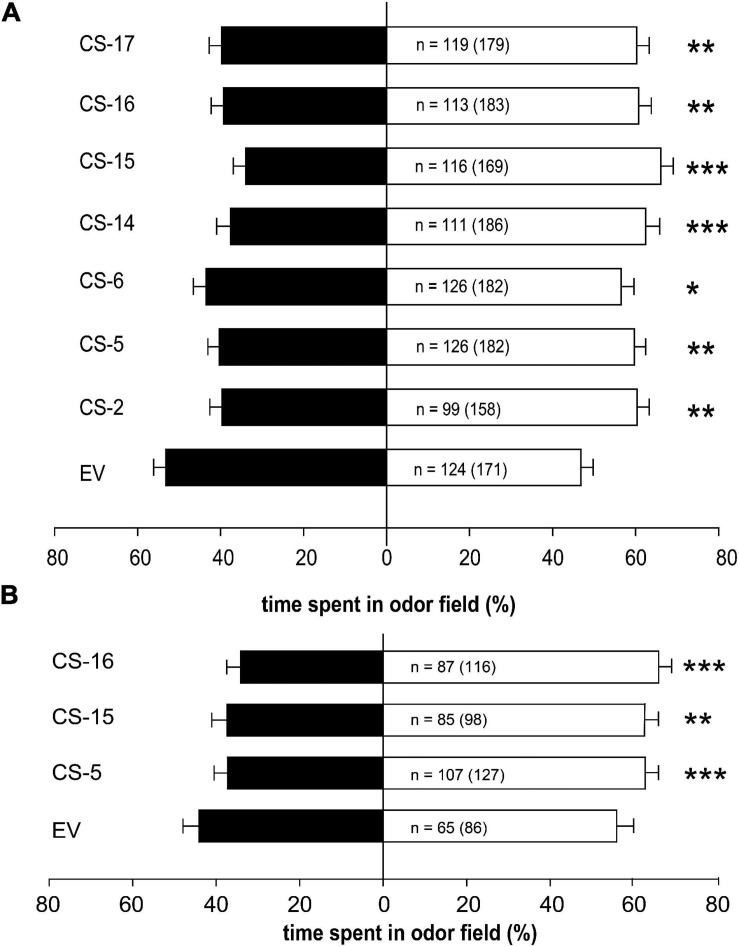
Responses of healthy **(A)** and *C*Las-infected **(B)** *D. citri*, tested in a four-arm-olfactometer, to volatiles emitted from flushes of EV and CS sweet orange lines. Black and white bars represent the overall percentage of time spent in plant and clean air odor sources, respectively. Error bars represent SD. For each comparison, the number of responding psyllids is indicated and the total number of insects tested is given between parentheses. Asterisks indicate significant differences between odor sources (*p < 0.05; **p < 0.001; ***p < 0.0001).

The response of *D. citri* to volatiles emitted from the transgenic orange lines was assessed in a four-arm olfactometer (Figure 6). Psyllids spent significantly less time in odor fields corresponding to volatiles from all CS lines than in clean air fields (Figure 6A). Highest significant effect was observed for odor field from line CS-15, in which the psyllids spent about 36% less time in CS fields in relation to EV, while the lowest effect was recorded for CS-6 odor field, in which psyllids expended a bit more than 18% less time than in that of EV. In the remaining CS lines, the reduction of time spent vs. EV was between 24 and 29%.

To find out whether *C*Las-infected psyllids were also deterred by CS volatiles, new behavioral assays were performed with three selected engineered lines showing different terpene accumulation profiles. A line with increased amount of (*E*)-β-caryophyllene (CS-15), a line with reduced content of orange leaf characteristic aroma monoterpenes (CS-16), and a line without changes in the accumulation of terpenes with respect to the controls (CS-5) were selected. Regardless of their volatile accumulation profile, infected psyllids spent significantly less time in odor fields from CS selected lines than in EV ones (Figure 6B), with reductions between 15% (CS-5 and -15) and 22% (CS-16).

It is interesting to note that time spent by psyllids in CS odor fields was lower than in EV fields (Figure 6), but percentages varied from one set of experiments (healthy psyllids) vs. the other (*C*Las-infected psyllids), which were performed along different months and then using different plant propagations. Then to ensure that CS lines are consistently less attractive to the psyllids and the effect of *C*Las infection on *D. citri* behavior, new statistical analysis was performed. When comparing the data from both experiments (Supplementary Figure 2), results indicated that just the genotype of the assayed lines was influencing *D. citri* response (*F* = 5.97, *p* = 0.0005), regardless of whether it was *C*Las infected or not (*F* = 2.24, *p* = 0.1348) and without interaction between these two factors (*F* = 1.22, *p* = 0.2999).

### Volatile Terpene Emission Profile of CS Leaves Is Highly Disturbed

**FIGURE 7:**
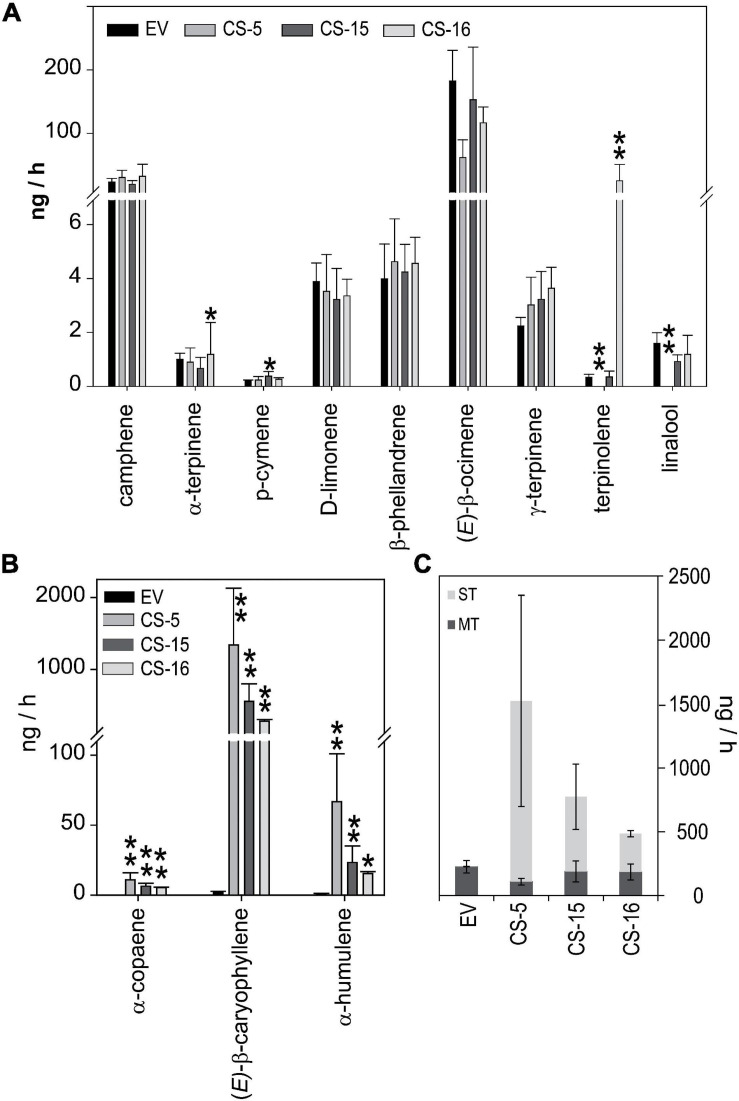
Quantitative measurement of terpene emission by flushes from EV and CS sweet orange lines. Volatiles were trapped on Tenax and eluted and analyzed by GC-MS as described in Section “Materials and Methods.” Monoterpenes **(A)** and sesquiterpenes **(B)** emitted by EV (black bars) and CS lines (gray lines). **(C)** Total monoterpenes (dark gray) and sesquiterpenes (light gray) emitted by the different genotypes. Data correspond to mean ± SD of three biological replicates. Asterisks indicate significant differences (*p < 0.05; **p < 0.01).

As *D. citri*, *C*Las infected or not, showed different responses to CS and EV lines that could not be directly related to variations in their volatile terpene content, the emission of these compounds by flushes of selected lines was analyzed. Again, lines CS-5, -15, and -16, plus one control EV line, were chosen. Total monoterpene emission of the CS lines did not differ much from EV lines with the exception of CS-5, which did not emit terpinolene and linalool (Figure 7A). However, the emission of terpinolene was more than 200-fold higher in CS-17 than in the other lines. Regarding sesquiterpenes, the only ones detected in our emission analyses were those produced by AtCS activity, namely, α-copaene, (*E*)-β-caryophyllene, and α-humulene (Figure 7B). The first one was not detected in EV volatile profiles, while the other two were emitted at a rate of 2 ng/h or lower. Emission rates of all three compounds were much higher in the CS engineered lines, at least 5, 279, and 15 ng/h for α-copaene, (*E*)-β-caryophyllene, and α-humulene, respectively. In CS lines, this boosts the emission of sesquiterpene compounds, which was between 100 and 300 times higher than that of controls, leading to an increase in total terpene emission (Figure 7C). Monoterpene emission was slightly reduced, although not to a significant extent. As a result, there was a radical shift in the profile of volatiles emitted by the CS lines: monoterpenes constituted 99% of the total emitted volatiles in EV lines, and this decreased to 36% in CS-16 and even to 7% in CS-5 (Figure 7C).

### *Diaphorina citri* Is Less Attracted to CS Engineered Sweet Orange Lines in No-Choice Assays

**FIGURE 8:**
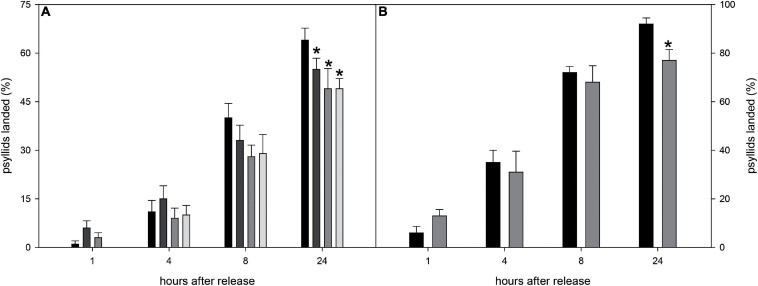
**(A)** Percentage of *D. citri* psyllids settled in CS-5 (dark light gray bars), CS-15 (medium gray bars), CS-16 (light gray bars), and EV (black bars) plants sprayed with kaolin to avoid visual cues 1, 4, 8, and 24 h after their release. **(B)** Percentage of *D. citri* psyllids settled in CS-15 (medium gray bars) and EV (black bars) plants 1, 4, 8, and 24 h after their release. For a given time, asterisks indicate significant differences between CS and EV lines (*p < 0.05). Error bars represent SD (*n* = 10 independent tests, 10 psyllids evaluated in each test).

To further evaluate the effect of the volatiles emitted by selected CS-lines on *D. citri* behavior, no-choice assays were performed. This kind of experiment may give a more realistic view of the response of psyllids, as they can move more freely than inside the olfactometer arena, and visual cues of the orange tree flushes can be added as an attractive parameter to the insect or avoided (with a kaolin spray). At least 99 responsive psyllids were evaluated (at 10 different days) for each genotype, thus circumventing any bias due to uncontrollable factors (atmospheric pressure, air relative humidity, light, etc.). Based on olfactometric and chemical results (Figures 6, 7), lines CS-5 and -15 (highly repellent and showing the most altered emission profile) and healthy psyllids (easier to culture and not-requiring qPCR to test *C*Las infection) were selected for these experiments. In order to avoid the effect of visual cues on this kind of assays, the plants were sprayed with kaolin. Each line was evaluated releasing 10 psyllids each day and scoring their location 1, 4, 8, and 24 h afterward. Eight hours after their release, the EV line contained at least 21% more insects than CS-lines, although the effect was not statistically significant (Figure 8A). At 24 h, the two tested CS-lines still harbored less *D. citri* individuals than the EV control line, with significant reductions of 14 and 23% (for CS-5 and -15, respectively).

Finally, the response of *D. citri* to a selected CS engineered line (CS-15, highest repellent effect, Figure 8A) was evaluated in a no-choice assay, but this time, the plants were not treated with kaolin, to take into account the attractiveness promoted by visual cues too. As before, 10 psyllids per plant were evaluated at 10 different days, and the number of psyllids that settled on the different lines was evaluated over 24 h (Figure 8B). At short time intervals (<8 h), only a slight non-significant reduction in the number of psyllids on CS lines was observed. After 24 h, however, the proportion of psyllids on the flushes was about 16% lower for CS than for EV line.

## Discussion

Huanglongbing is a vector-transmitted bacterial disease seriously threatening citriculture worldwide. Despite the giant research efforts, a cure for this disease has not yet been found, and control is based on avoiding HLB-infection by reducing the amount of bacterial inoculum (by removing infected trees and replanting with healthy certified plant material) and reducing the population of vector psyllids (mainly with chemical insecticides). Huge research efforts on new management strategies to control HLB are still ongoing from many different angles, although, up to date, all of them showed limited and/or variable efficacy (reviewed in Khan et al., 2014; Fancelli et al., 2018; National Academies of Sciences, Engineering, and Medicine, 2018; Cochrane and Shade, 2019). Meanwhile, HLB is spreading, killing millions of trees, contaminating the environment, and causing billion dollar economic losses around the world. Besides, consistent resistance traits have not yet been identified in *Citrus*-related germplasm, hampering breeding approaches, which are already practically unattainable in citrus due to complex genetics and reproductive characteristics. In view of such a devastating scenario, it has been proposed that the only sustainable solution to control HLB is the generation of genetically modified citrus cultivars resistant either to the causal bacterium, to the transmitting vectors, or to both (Duran-Vila et al., 2014; Mccollum and Baldwin, 2016; Killiny et al., 2018; National Academies of Sciences, Engineering, and Medicine, 2018; Cochrane and Shade, 2019; Wang, 2019). Altering the emission of host volatile cues in order to modify pest/pathogen–plant–natural enemies’ interactions is an environmental-friendly strategy to achieve plant protection to pests and pathogens. Due to the non-toxic nature of these semiochemicals, research in this field has increased in the last decade, numerous transgenic plants have been generated, and their ability to resist or tolerate infestation/infection to different pests and pathogens was demonstrated in laboratory assays. For example, a wheat cultivar engineered to release (*E*)-β-farnesene repelled three species of cereal aphids in laboratory tests, though not in small preliminary field trials (Bruce et al., 2015).

The importance of olfactory cues in host plant finding by *D. citri* is well documented (Rouseff et al., 2008; Patt and Sétamou, 2010; Zaka et al., 2010; Mann et al., 2012; Robbins et al., 2012; Silva et al., 2016; Fancelli et al., 2018). Moreover, it has been proposed that the emitted volatile profile from young flushes may play a pivotal role in determining *D. citri* host suitability (Westbrook et al., 2011). We have reported previously that the volatile sesquiterpene β-caryophyllene, when emitted from a chemical dispenser or transgenic *Arabidopsis* plants, exerted a repellent effect on *D. citri* (Alquézar et al., 2017b). Thus, constitutive β-caryophyllene emission from citrus trees could possibly make them repellent or at least non-attractive to the psyllid. To explore this possibility, “Valencia” sweet orange plants engineered to constitutively express a β*-CARYOPHYLLENE SYNTHASE* (CS lines) and with no obvious phenotypic differences in relation to EV control plants were produced. As new shoots are the most attractive plant material to *D. citri* for feeding and oviposition (Cifuentes-Arenas et al., 2018), all subsequent characterizations of the CS lines were performed on leaves of this phenological stage, and to evaluate psyllid responses to them, adult psyllids were employed because of their flying ability and thus their relevance for HLB dispersal.

In all analyzed lines, monoterpenes were the predominant volatile compounds in leaf extracts, constituting 63–83% of the total mono- and sesquiterpenoids detected, as previously described for sweet orange leaves (Azam et al., 2013; Alquézar et al., 2017a). Despite this, changes in the content of certain terpenes were detected in most lines. In future works, to gain insight into orange volatile terpene biosynthetic pathway regulation, it will be interesting to determine whether different volatile profiles identified among CS lines are due to differences in substrate availability to terpene synthesis or to different induced expression/activity changes of other sesqui- or monoterpene synthases. A boost in β-caryophyllene accumulation was detected only in lines CS-6, -15, and -17. However, when flushes from CS lines were challenged with *D. citri*, they all were less attractive to the psyllid.

When volatile emission of the transgenic lines was investigated, important differences were found in relation to the composition of mono- and sesquiterpenes. For example, β-caryophyllene emission by the three analyzed CS lines was increased at least 190-fold compared with the EV line, and to a higher extent in CS-5, which in leaf extracts had the same amount of this sesquiterpene as the EV line. Differences in stored and emitted volatile profiles have been described for citrus and other plants before (LLusia and Peñuelas, 2000; Ormen et al., 2011; Fares et al., 2012; Alquézar et al., 2017a). The release of volatiles from plant tissues depends on many factors, such as the physicochemical characteristics of the terpenoids, the tissue in which they are produced, and the presence of biotic and abiotic stresses, among others (Widhalm et al., 2015). As all CS lines were grown and analyzed under controlled conditions, differences in VOC emission between CS-5 and the other two lines may be related to other processes involved in the release of these compounds from their site of biosynthesis, such as vesicular transport, interorganellar membrane hemifusion, carrier proteins, or transporters, among others (Ting et al., 2015; Widhalm et al., 2015; Pierman et al., 2017). It will also be interesting to determine if different volatile profiles identified among CS lines are due to differences in substrate availability to terpene synthesis or to different induced expression/activity changes of other sesqui- or monoterpene synthases. As the enormous increase in the emission of sesquiterpenes in CS lines was not accompanied by changes of similar magnitude in that of monoterpenes, there was a radical shift in the emission profile, with sesquiterpenes becoming the dominant volatiles. When fighting a pest, maximum disturbance of the volatile emission profile is probably a desirable trait because herbivore host location is mediated not by the presence of individual compounds, but by specific blends in which certain compounds are present in specific proportions (Bruce and Pickett, 2011; Reinecke and Hilker, 2014). For example, a highly attractive blend to the orange wheat blossom midge turned it into neutral when the dose of one of the components was increased threefold (Birkett et al., 2004). Regarding β-caryophyllene, in CS lines, it contributed from 60 to more than 80% of total volatiles, while in EV, as previously described (Stelinski and Tiwari, 2013; Martini et al., 2018; Patt et al., 2018), it constituted less than 1% of total volatiles emitted by healthy Valencia orange flushes.

Lines CS-5, -15, and -16, which exhibited great changes in stored and emitted volatile profiles (especially in relation to β-caryophyllene), were also highly deterrent to *D. citri* in olfactometric assays. Although this kind of analysis allows comparisons of different odor sources under very well-controlled standardized conditions, it only enables the insects to move horizontally. As *D. citri* moves, more than walking, by short flights (Stelinski and Tiwari, 2013), its response to CS-5, -15, and -16 volatiles was re-evaluated in a more “realistic” way by confining them into big boxes (of about 50 million times psyllid volume) and observing their behavior toward orange flushes. Less psyllids landed on β-caryophyllene over-emitting leaves than on EV controls, independent of the presence or not of visual cues. Kaolin is an aluminum silicate that, when sprayed on the plants, creates a non-abrasive white particle film over them. White-kaolin color disrupts plant visual cues, turning them unrecognizable to the host. When combining a CS line with kaolin spraying, reductions of up to 61.11 and 46.74% in the number of settled psyllids were observed in relation to untreated EV flushes at 8 and 24 h, respectively. Thus, although field assays and agronomical characterization are required, a new “Valencia” sweet orange genotype, well recognized worldwide for its superior organoleptic characteristics and grown extensively in most citrus countries, was developed in the present paper. This new Valencia β-caryophyllene over-emitting genotype is repellent to the main HLB-transmitting vector, *D. citri*, as demonstrated in four-arm olfactometric tests. The confinement of insects can artificially induce positive responses to species or genotypes that may not hold up in the field (Driesche and Murray, 2004). In fact, settling of confined *D. citri* on non-host plants such as *Arabidopsis*, cotton, guava, tomato, and azalea (Hall et al., 2008; Ruan et al., 2015; Alquézar et al., 2017b) and even on inert material with visual cues (Paris et al., 2017) has been reported before. From no-choice experiments conducted in this work, it is clear that CS genotypes were less preferred by the psyllid than the controls, but we do not know how this will be in the field. For instance, under our experimental conditions, kaolin treatment reduced psyllid landing on host plants by 35–40%, but in the field, the reduction was 50–96% (Hall et al., 2007; Miranda et al., 2018; Ramírez-Godoy et al., 2018).

## Conclusion

In conclusion, by inducing β-caryophyllene emission, we have turned an elite sweet orange cultivar, usually attractive to *D. citri* in the field, into a repellent/non-attractive genotype. Combining the use of this new genotype with kaolin treatment, which reduces psyllid landing in the field, would disrupt both visual and olfactory cues used by the psyllid, and thus, it may impede host finding. Moreover, although the palatability of CS lines by the psyllids has not yet been determined, kaolin treatment also reduces *D. citri* feeding on phloem (Miranda et al., 2018), decreasing even more the chances of HLB-infection. Additionally, to completely divert the psyllids from the highly attractant commercial orange crop, companion plant species attractive to the psyllids could be planted nearby. The use of this push/pull strategy combining repellent and attractive plant species has been successfully used to control cereal pests (Khan et al., 1997; Pickett et al., 2010, 2014; Kortbeek et al., 2019). In citrus orchards, when the highly *D. citri* attractive rutaceous *M. paniculata* ornamental is used as border trap plant, the number of psyllids inside the citrus orchard is greatly reduced, especially if the trap plants are periodically treated with insecticides to kill the insects (Tomaseto et al., 2019). Another advantage of *M. paniculata* is that because of the low titers and transient infection by HLB-associated bacteria and the low, epidemiologically irrelevant, back-transmission rates to citrus by *D. citri*, it cannot be considered an inoculum reservoir, but is a dead-end host (Cifuentes-Arenas et al., 2019). The performance of this push (CS lines plus kaolin)/pull (attractive *M. paniculata* plants plus insecticide treatments) system to deter the psyllids and stop HLB dispersal will be evaluated in a more than 20-ha field experiment, which has recently been planted. The combination of avoiding visual and olfactory host cues with a trap and kill border crop is expected to decrease HLB incidence, while greatly reducing the economic and environmental costs due to the large reduction in the amount of insecticides used to control psyllid populations (Miranda et al., 2018; Tomaseto et al., 2019).

## Data Availability Statement

The original contributions presented in the study are included in the article/Supplementary Material. Further inquiries can be directed to the corresponding author/s.

## Author Contributions

BA drafted the manuscript and contributed to all the experiments and analyses. MM and HV performed insect behavioral assays and statistical analyses. RM, MV, and RS performed VOC analyses. MS performed qRT-PCR and southern analyses. VM, NW, and MA generated transgenic citrus. H-MT and HB contributed to construct generation. LP and HB provided tools and ideas. LP conceived, designed, and supervised the study, and approved the final version of the manuscript. All authors reviewed the manuscript.

## Conflict of Interest

The authors declare that the research was conducted in the absence of any commercial or financial relationships that could be construed as a potential conflict of interest.

## Supplementary Material

Supplementary Figure 1 **(A)** Schematic diagram of the T-DNA region of the PinPlus vector used for plant transformation in order to induce β-caryophyllene production. LB and RB, left and right T-DNA borders, respectively; p35S, CaMV 35S promoter; CS, genomic clone of *AtTPS21* (*At5g23960*); tRbcS, terminator region of the small subunit of the ribulose *1*,5-bisphosphate carboxylase gene from *Arabidopsis*; KanR, neomycin phosphotransferase II (*nptII*) selectable marker gene conferring kanamycin resistance; NOSt and NOSp, nopaline synthase terminator and promoter sequences, respectively. The transcription orientation for each cassette is indicated by arrows on the promoter and coding sequences. **(B)** Southern blot analysis to test the integrity of the inserted T-DNA using genomic DNA treated with *Nhe*I and *Sac*I restriction enzymes (NEB). **(C)** Southern blot analysis indicating the loci number of the inserted T-DNA from genomic DNA treated with *Nhe*I. M. Molecular weight marker II (Roche Applied Biosciences) and digoxigenin-11-dUTP (Roche Applied Biosciences)-labeled *nptII* probe coding sequence were used in both **(B)** and **(C)**.

Supplementary Figure 2 Percentage (average ± SD) of time spent in plant odor sources from flushes of EV and CS sweet orange lines by healthy (black bars) and *C*Las-infected (gray bars) *D. citri*, tested in a 4-arm-olfactometer. The overall mean time (±SD) spent that psyllids (regardless of their infection status) in odor fields from each genotype is represented by red bars. Asterisks indicate significant differences between EV and CS lines (^∗^*p* < 0.001). Between lines, no significant differences were found when the response of healthy and infected psyllids was compared.

Supplementary Figure 3 **(A)** General view of the device developed to evaluate in no-choice tests the effect of plant volatiles on *Diaphorina citri*. Details of the central processing unit (CPU) fan cooler **(B)**, the digital potentiometer **(C)**, the psyllids release platform **(D)**, and the aspect of the flushes used in these experiments **(E)**.
