# Supplementary figures and images for: Engineered Orange Ectopically Expressing the Arabidopsis β-Caryophyllene Synthase Is Not Attractive to Diaphorina citri, the Vector of the Bacterial Pathogen Associated to Huanglongbing
Source: Front Plant Sci. 2021 Mar 2;12:641457. doi: 10.3389/fpls.2021.641457 (PMC7982956; doi:10.3389/fpls.2021.641457)

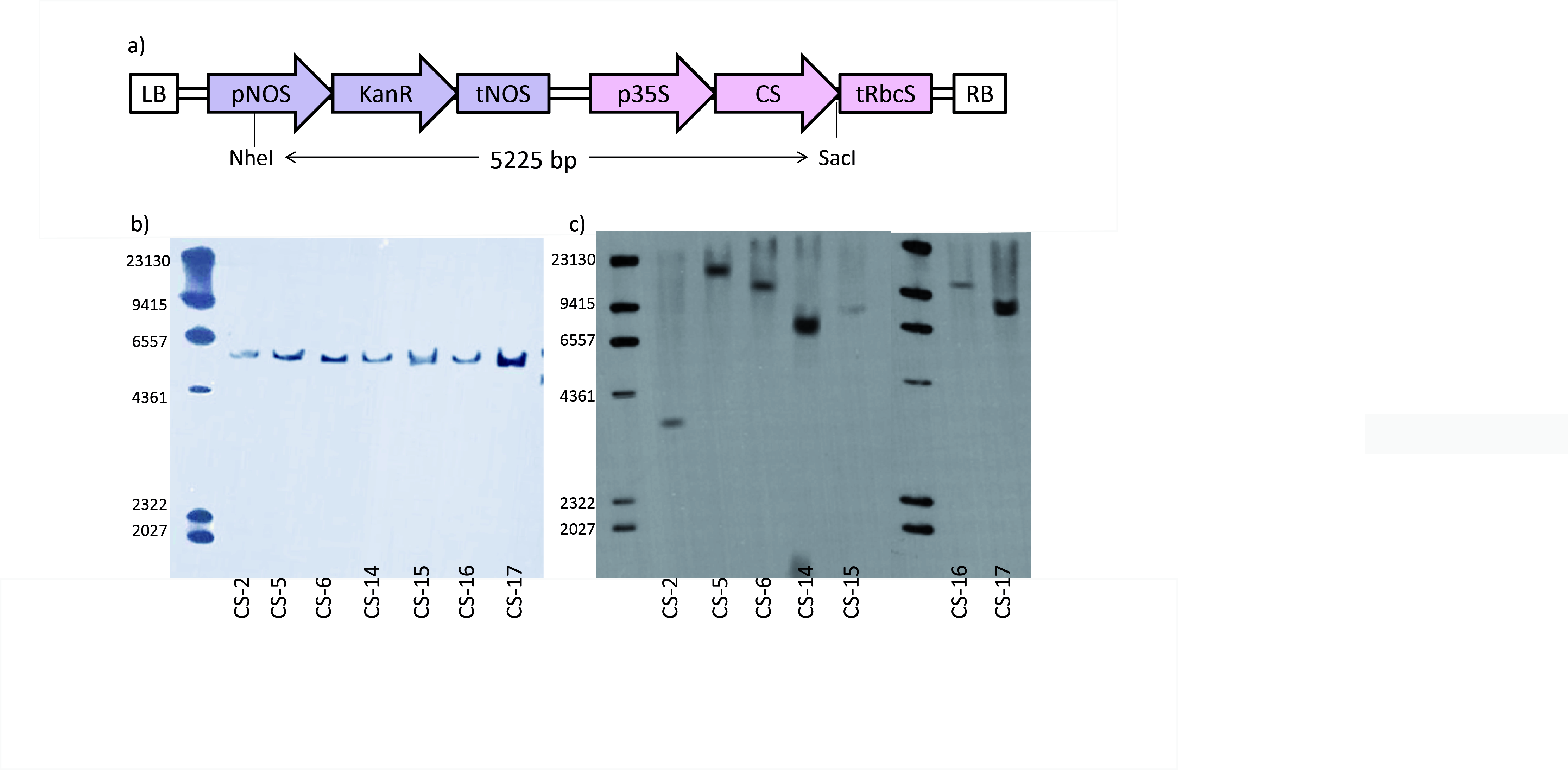

Supplement: Supplementary Figure 1 — (A) Schematic diagram of the T-DNA region of the PinPlus vector used for plant transformation in order to induce β-caryophyllene production. LB and RB, left and right T-DNA borders, respectively; p35S, CaMV 35S promoter; CS, genomic clone of AtTPS21 (At5g23960); tRbcS, terminator region of the small subunit of the ribulose 1,5-bisphosphate carboxylase gene from Arabidopsis; KanR, neomycin phosphotransferase II (nptII) selectable marker gene conferring kanamycin resistance; NOSt and NOSp, nopaline synthase terminator and promoter sequences, respectively. The transcription orientation for each cassette is indicated by arrows on the promoter and coding sequences. (B) Southern blot analysis to test the integrity of the inserted T-DNA using genomic DNA treated with NheI and SacI restriction enzymes (NEB). (C) Southern blot analysis indicating the loci number of the inserted T-DNA from genomic DNA treated with NheI. M. Molecular weight marker II (Roche Applied Biosciences) and digoxigenin-11-dUTP (Roche Applied Biosciences)-labeled nptII probe coding sequence were used in both (B) and (C). [file Image_1.TIF]

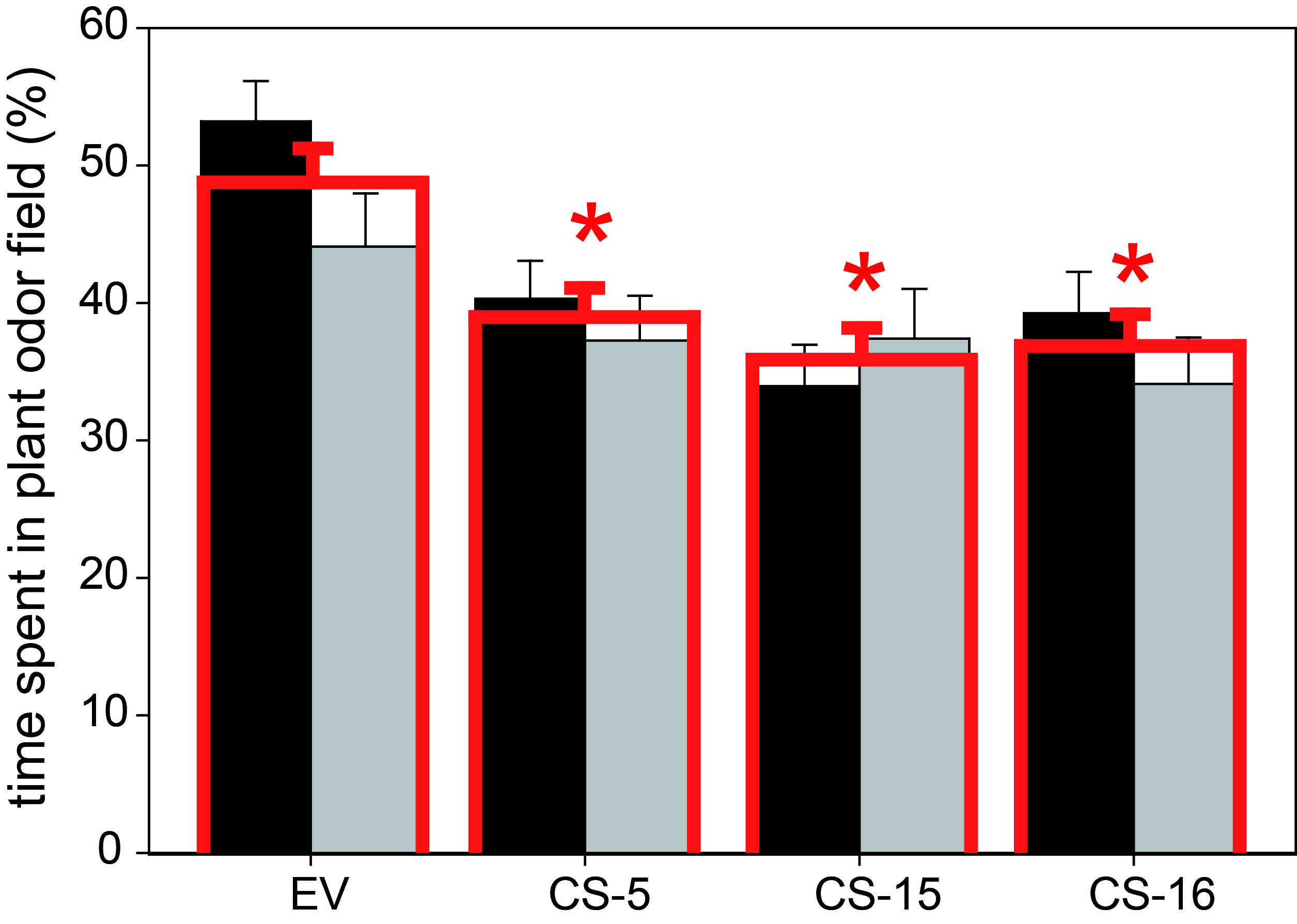

Supplement: Supplementary Figure 2 — Percentage (average ± SD) of time spent in plant odor sources from flushes of EV and CS sweet orange lines by healthy (black bars) and CLas-infected (gray bars) D. citri, tested in a 4-arm-olfactometer. The overall mean time (±SD) spent that psyllids (regardless of their infection status) in odor fields from each genotype is represented by red bars. Asterisks indicate significant differences between EV and CS lines (∗p < 0.001). Between lines, no significant differences were found when the response of healthy and infected psyllids was compared. [file Image_2.TIF]

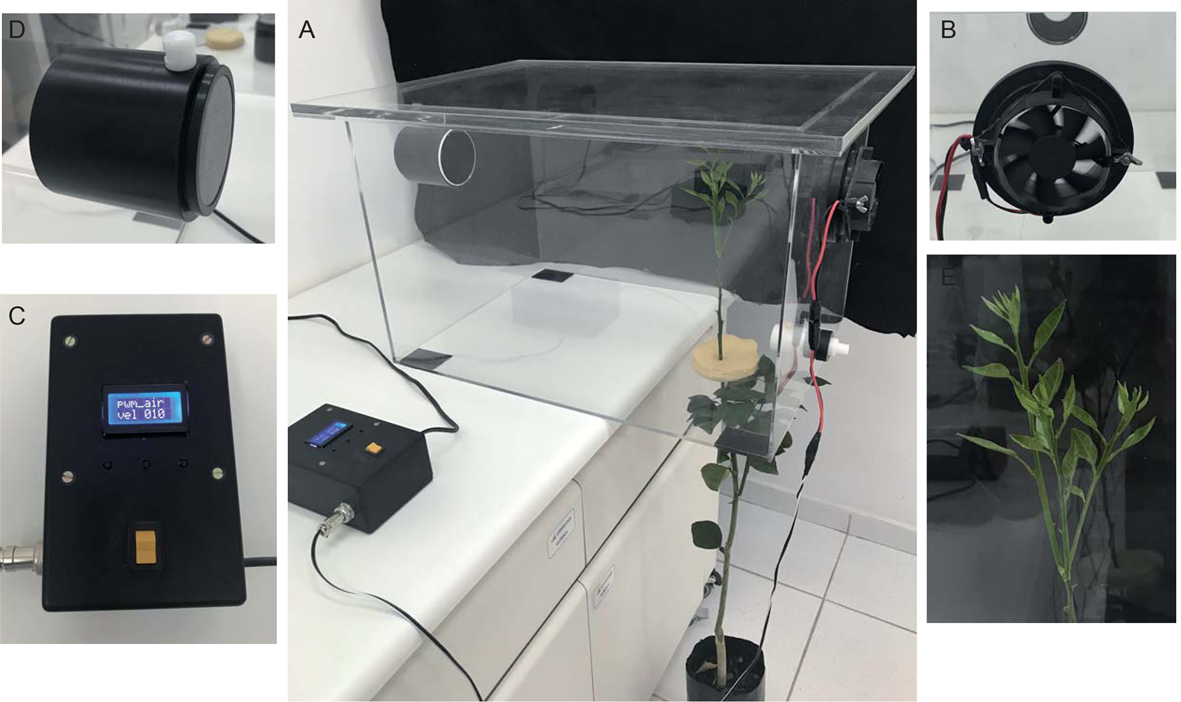

Supplement: Supplementary Figure 3 — (A) General view of the device developed to evaluate in no-choice tests the effect of plant volatiles on Diaphorina citri. Details of the central processing unit (CPU) fan cooler (B), the digital potentiometer (C), the psyllids release platform (D), and the aspect of the flushes used in these experiments (E). [file Image_3.TIF]
